# *rac*-3-*exo*-Ammonio-7-*anti*-carb­oxy­tricyclo­[2.2.1.0.^2,6^]heptane-3-*endo*-carboxyl­ate monohydrate

**DOI:** 10.1107/S1600536812016236

**Published:** 2012-04-21

**Authors:** Graham Smith, Urs D. Wermuth, Ian D. Jenkins

**Affiliations:** a Science and Engineering Faculty, Queensland University of Technology, GPO Box 2434, Brisbane, Queensland 4001, Australia; b School of Biomolecular and Physical Sciences, Griffith University, Nathan, Queensland, 4111, Australia; c Eskitis Institute, Griffith University, Nathan, Queensland, 4111, Australia

## Abstract

The racemic title compound, C_9_H_11_NO_4_·H_2_O, a tricyclic rearranged amino­norbornane dicarb­oxy­lic acid, is a conformationally rigid analogue of glutamic acid and exists as an ammonium-carboxyl­ate zwitterion, with the bridghead carb­oxy­lic acid group *anti*-related. In the crystal, N—H⋯O and O—H⋯O hydrogen bonds involving the ammonium, carb­oxy­lic acid and water donor groups with both water and carboxyl O-atom acceptors give a three-dimensional framework structure.

## Related literature

For background to G-protein receptors, see: Liu & Doller (2011 ▶). For the Strecher and Bucherer–Bergs reactions, see: Strecher (1850 ▶); Bucherer & Steiner (1934 ▶). For the synthesis of amino­norbornane carb­oxy­lic acids, see: Apgar & Ludwig (1972 ▶); Tager & Christensen (1972 ▶); Wermuth (1995 ▶). For the chemistry of hydantoins, see: Avendaño López & González Trigo (1985 ▶). For the structure of a similar monocarb­oxy­lic acid tricyclic cage compound, see: Fortier *et al.* (1979 ▶). For graph-set analysis, see: Etter *et al.* (1990 ▶).
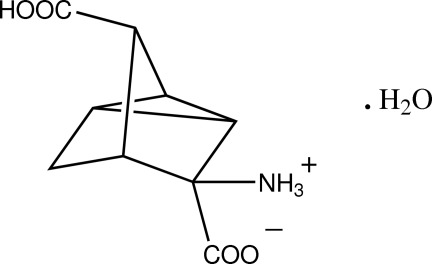


## Experimental

### 

#### Crystal data

C_9_H_11_NO_4_·H_2_O
*M* *_r_* = 215.20
Monoclinic, 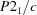

*a* = 7.7565 (2) Å
*b* = 11.4103 (2) Å
*c* = 10.3339 (3) Å
β = 94.888 (2)°
*V* = 911.27 (4) Å^3^
*Z* = 4
Mo *K*α radiation
μ = 0.13 mm^−1^
*T* = 223 K
0.30 × 0.30 × 0.15 mm

#### Data collection

Oxford Diffraction Gemini-S Ultra CCD-detector diffractometer
Absorption correction: multi-scan (*CrysAlis PRO*; Oxford Diffraction, 2010 ▶) *T*_min_ = 0.990, *T*_max_ = 1.000
7572 measured reflections
2128 independent reflections
1589 reflections with *I* > 2σ(*I*)
*R*_int_ = 0.026

#### Refinement

*R*[*F* ^2^ > 2σ(*F* ^2^)] = 0.038
*wR*(*F* ^2^) = 0.091
*S* = 0.97
2128 reflections
160 parameters
H atoms treated by a mixture of independent and constrained refinement
Δρ_max_ = 0.31 e Å^−3^
Δρ_min_ = −0.25 e Å^−3^

### 

Data collection: *CrysAlis PRO* (Oxford Diffraction, 2010 ▶); cell refinement: *CrysAlis PRO*; data reduction: *CrysAlis PRO*; program(s) used to solve structure: *SIR92* (Altomare *et al.*, 1994 ▶); program(s) used to refine structure: *SHELXL97* (Sheldrick, 2008 ▶) within *WinGX* (Farrugia, 1999 ▶); molecular graphics: *PLATON* (Spek, 2009 ▶); software used to prepare material for publication: *PLATON*.

## Supplementary Material

Crystal structure: contains datablock(s) global, I. DOI: 10.1107/S1600536812016236/lh5454sup1.cif

Structure factors: contains datablock(s) I. DOI: 10.1107/S1600536812016236/lh5454Isup2.hkl

Supplementary material file. DOI: 10.1107/S1600536812016236/lh5454Isup3.cml

Additional supplementary materials:  crystallographic information; 3D view; checkCIF report

## Figures and Tables

**Table 1:** Hydrogen-bond geometry (Å, °)

*D*—H⋯*A*	*D*—H	H⋯*A*	*D*⋯*A*	*D*—H⋯*A*
O1*W*—H11*W*⋯O71^i^	0.90 (2)	2.02 (2)	2.9161 (16)	176 (2)
O1*W*—H12*W*⋯O31^ii^	0.93 (2)	1.77 (2)	2.6792 (16)	168 (2)
N31—H31*A*⋯O31^ii^	0.94 (2)	1.87 (2)	2.7712 (17)	161 (2)
N31—H31*B*⋯O71^iii^	0.91 (2)	2.29 (2)	3.1261 (17)	153.0 (15)
N31—H31*B*⋯O72^iii^	0.91 (2)	2.27 (2)	3.0720 (17)	147.6 (15)
N31—H31*C*⋯O32^iv^	0.874 (18)	1.925 (17)	2.7769 (16)	164.4 (17)
O72—H72⋯O1*W*	0.94 (3)	1.60 (3)	2.5282 (16)	174 (3)

Symmetry codes: (i) 
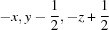
; (ii) 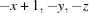; (iii) 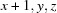; (iv) 
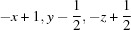
.

**Fig. 1.**
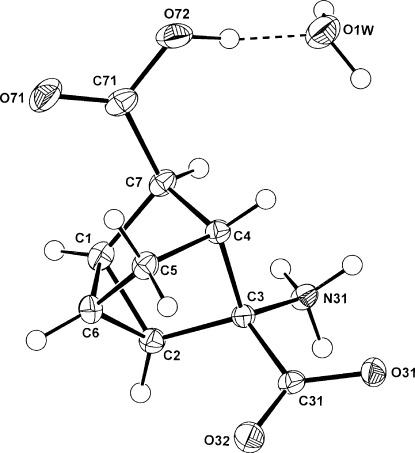
Molecular configuration and atom naming scheme of the zwitterionic title compound (I). The inter-species hydrogen bond is shown as a dashed line and displacement ellipsoids are drawn at the 40% probability level.

**Fig. 2.**
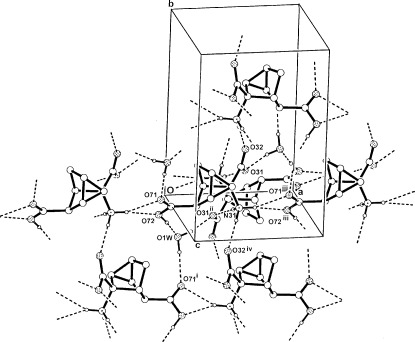
Hydrogen-bonding (shown as dashed lines) in the three-dimensionlal structure of the title compound, viewed approximately along the c axis. The symmetry codes are as in Table 1.

**Fig. 3.**
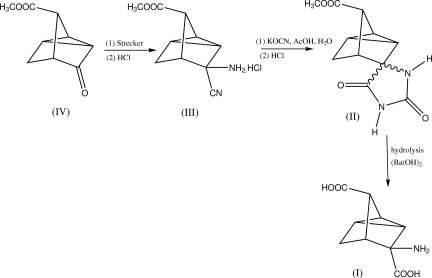
The reaction scheme for the synthesis of the title compound.

## supplementary crystallographic information

### Comment

G-protein-coupled receptors (GPCRs) constitute a superfamily of proteins whose main function is to convert extracellular stimuli into intracellular signals (Liu & Doller, 2011). Metabotropic glutamate (mGu) receptors belong to the class C GPCR group and are activated by *L*-glutamate. The title compound, C_9_H_11_NO_4_. H_2_O (I), is a hydrated tricyclic rearranged aminonorbornane dicarboxylic acid cage compound which is a conformationally rigid analogue of glutamic acid, and was synthesized as a potential ligand for metabotropic glutamate receptors in order to explore the requirements for activity at these receptors (Wermuth, 1995). For the synthesis of amino-substituted norbornane carboxylic acids, see also Tager & Christensen (1972) and Apgar & Ludwig (1972).

The title compound exists as an ammonium carboxylate zwitterion with the C3 carboxylate group *endo*-oriented (Fig. 1). Note that the stereochemical assignment of *exo* and *endo* on such nortricyclic systems is somewhat arbitrary and depends on how the system is drawn. The carboxylic acid group at C7 in (I) is *exo* and has the acid H-atom (H72) *anti*-located, forming a hydrogen bond with the water molecule of solvation (Table 1). This water molecule gives intermolecular hydrogen-bonding associations with carboxyl O-atom acceptors, while the ammonium group also forms four hydrogen bonds with carboxyl O-atom acceptors. These include a symmetric cyclic N—H···O,*O*' head-to-tail association [graph set *R*^2^_1_(4) (Etter *et al.*, 1990)] which links the molecules along (100). Overall, a three-dimensional framework structure is formed (Fig. 2).

The structures of similar tricyclic norbornane compounds are rare in the crystallographic literature. The nortricyclic keto acid which served as the precursor to (IV) in the synthesis of (I) (Fig. 3) is known (Fortier *et al.*, 1979).

### Experimental

The title compound (I) was synthesized (Wermuth, 1995) by the hydrolysis with Ba(OH)_2_ of the diasteroisomeric hydantoin mixture (II), which was obtained by a Read synthesis (Avendaño López & González Trigo, 1985) performed on the nortricyclic keto-ester (IV) (Fig. 3). Briefly, a Strecker aminonitrile (Streker, 1850) is formed in the usual manner (50% yield) and this was converted to a hydrochloride (III) and reacted with KOCN in an acetic acid–water mixture at 273K for 1 h followed by the addition of conc. HCl and heating for a further 15 min at 273K. The product was a diastereomeric mixture of hydantoins in 49% yield after recrystallization from 50% aqueous ethanol. The stereochemistry of the amino acid moiety is the inverse (carboxylic acid group *exo*) of that normally formed in the Bucherer-Bergs reaction (Bucherer & Steiner, 1934). The colourless product obtained gave an elemental analysis consistent with a 0.25 hydrate but recrystallization from various solvents gave no crystals suitable for X-ray analysis. However, colourless plates of a monohydrate (I) were obtained from the attempted reaction of this partial hydrate with picrylsulfonic acid in 80% propan-2-ol-water and a specimen suitable for the X-ray analysis was cleaved from a larger crystal.

### Refinement

Ammonium and water H atoms were located in a difference Fourier map and both positional and isotropic displacenment parameters were refined. Other H atoms were included in the refinement at calculated positions [C—H = 0.97–0.98 Å] with *U*_iso_(H) = 1.2*U*_eq_(C), using a riding-model approximation. The relative configuration of the molecule described for (I) is C1(*R*), C2(*S*), C3(*R*), C4(*R*), C6(*S*), C7(*R*).

### Crystal data

**Table:**

C_9_H_11_NO_4_·H_2_O	*F*(000) = 456
*M**_r_* = 215.20	*D*_x_ = 1.569 Mg m^−^^3^
Monoclinic, *P*2_1_/*c*	Mo *K*α radiation, λ = 0.71073 Å
Hall symbol: -P 2ybc	Cell parameters from 3396 reflections
*a* = 7.7565 (2) Å	θ = 3.2–28.6°
*b* = 11.4103 (2) Å	µ = 0.13 mm^−^^1^
*c* = 10.3339 (3) Å	*T* = 223 K
β = 94.888 (2)°	Plate, colourless
*V* = 911.27 (4) Å^3^	0.30 × 0.30 × 0.15 mm
*Z* = 4	

### Data collection

**Table:**

Oxford Diffraction Gemini-S Ultra CCD-detector diffractometer	2128 independent reflections
Radiation source: Enhance (Mo) X-ray source	1589 reflections with *I* > 2σ(*I*)
Graphite monochromator	*R*_int_ = 0.026
Detector resolution: 16.077 pixels mm^-1^	θ_max_ = 28.6°, θ_min_ = 3.2°
ω scans	*h* = −9→10
Absorption correction: multi-scan (*CrysAlis PRO*; Oxford Diffraction, 2010)	*k* = −15→15
*T*_min_ = 0.990, *T*_max_ = 1.000	*l* = −13→13
7572 measured reflections	

### Refinement

**Table:**

Refinement on *F*^2^	Primary atom site location: structure-invariant direct methods
Least-squares matrix: full	Secondary atom site location: difference Fourier map
*R*[*F*^2^ > 2σ(*F*^2^)] = 0.038	Hydrogen site location: inferred from neighbouring sites
*wR*(*F*^2^) = 0.091	H atoms treated by a mixture of independent and constrained refinement
*S* = 0.97	*w* = 1/[σ^2^(*F*_o_^2^) + (0.0525*P*)^2^ + 0.0562*P*] where *P* = (*F*_o_^2^ + 2*F*_c_^2^)/3
2128 reflections	(Δ/σ)_max_ = 0.001
160 parameters	Δρ_max_ = 0.31 e Å^−^^3^
0 restraints	Δρ_min_ = −0.25 e Å^−^^3^

### Special details

**Table:**

Geometry. Bond distances, angles *etc*. have been calculated using the rounded fractional coordinates. All su's are estimated from the variances of the (full) variance-covariance matrix. The cell e.s.d.'s are taken into account in the estimation of distances, angles and torsion angles
Refinement. Refinement of *F*^2^ against ALL reflections. The weighted *R*-factor *wR* and goodness of fit *S* are based on *F*^2^, conventional *R*-factors *R* are based on *F*, with *F* set to zero for negative *F*^2^. The threshold expression of *F*^2^ > σ(*F*^2^) is used only for calculating *R*-factors(gt) *etc*. and is not relevant to the choice of reflections for refinement. *R*-factors based on *F*^2^ are statistically about twice as large as those based on *F*, and *R*- factors based on ALL data will be even larger.

### Fractional atomic coordinates and isotropic or equivalent isotropic displacement parameters (Å^2^)

**Table:**

	*x*	*y*	*z*	*U*_iso_*/*U*_eq_	
O31	0.60834 (14)	0.12616 (8)	0.03042 (10)	0.0217 (3)	
O32	0.57555 (15)	0.27409 (8)	0.16495 (11)	0.0291 (4)	
O71	−0.10727 (14)	0.05736 (9)	0.32326 (12)	0.0311 (4)	
O72	−0.08102 (14)	−0.06274 (9)	0.16170 (11)	0.0273 (3)	
N31	0.53370 (17)	−0.03193 (10)	0.20183 (13)	0.0166 (3)	
C1	0.26343 (19)	0.07189 (12)	0.39029 (14)	0.0190 (4)	
C2	0.43453 (18)	0.12186 (11)	0.35275 (13)	0.0165 (4)	
C3	0.45247 (17)	0.08675 (11)	0.21340 (13)	0.0142 (4)	
C4	0.25626 (18)	0.08423 (11)	0.16738 (13)	0.0152 (4)	
C5	0.19989 (19)	0.20682 (11)	0.20992 (13)	0.0176 (4)	
C6	0.27559 (19)	0.19863 (12)	0.35020 (14)	0.0184 (4)	
C7	0.18333 (18)	0.00565 (12)	0.27376 (13)	0.0171 (4)	
C31	0.55486 (18)	0.17107 (11)	0.13073 (14)	0.0163 (4)	
C71	−0.01187 (19)	0.00291 (12)	0.25744 (15)	0.0210 (4)	
O1W	0.10015 (16)	−0.21234 (11)	0.05407 (12)	0.0312 (4)	
H1	0.24590	0.04870	0.47950	0.0230*	
H2	0.53470	0.13290	0.41600	0.0200*	
H4	0.22510	0.06230	0.07670	0.0180*	
H5A	0.25210	0.26910	0.16270	0.0210*	
H5B	0.07510	0.21590	0.20280	0.0210*	
H6	0.26610	0.26310	0.41190	0.0220*	
H7	0.23080	−0.07380	0.27130	0.0210*	
H31A	0.509 (3)	−0.0598 (15)	0.117 (2)	0.033 (5)*	
H31B	0.650 (3)	−0.0273 (14)	0.2222 (17)	0.028 (5)*	
H31C	0.501 (2)	−0.0843 (15)	0.2564 (18)	0.024 (4)*	
H72	−0.007 (4)	−0.115 (2)	0.124 (2)	0.063 (7)*	
H11W	0.108 (3)	−0.283 (2)	0.092 (2)	0.063 (7)*	
H12W	0.207 (3)	−0.1929 (19)	0.026 (2)	0.054 (6)*	

### Atomic displacement parameters (Å^2^)

**Table:**

	*U*^11^	*U*^22^	*U*^33^	*U*^12^	*U*^13^	*U*^23^
O31	0.0263 (6)	0.0193 (5)	0.0209 (5)	−0.0048 (4)	0.0095 (4)	−0.0035 (4)
O32	0.0346 (7)	0.0157 (5)	0.0393 (7)	−0.0075 (5)	0.0158 (5)	−0.0080 (5)
O71	0.0185 (6)	0.0331 (6)	0.0433 (7)	0.0021 (5)	0.0119 (5)	−0.0028 (5)
O72	0.0153 (5)	0.0251 (5)	0.0410 (7)	−0.0032 (5)	0.0002 (5)	−0.0035 (5)
N31	0.0143 (6)	0.0135 (5)	0.0223 (7)	0.0010 (5)	0.0038 (5)	0.0020 (5)
C1	0.0167 (7)	0.0230 (7)	0.0175 (7)	0.0017 (6)	0.0032 (6)	0.0026 (5)
C2	0.0156 (7)	0.0182 (6)	0.0157 (7)	−0.0008 (6)	0.0008 (5)	−0.0003 (5)
C3	0.0126 (7)	0.0126 (6)	0.0176 (7)	0.0006 (5)	0.0020 (5)	−0.0021 (5)
C4	0.0135 (7)	0.0164 (6)	0.0156 (7)	−0.0001 (5)	0.0008 (5)	−0.0002 (5)
C5	0.0167 (7)	0.0160 (6)	0.0203 (7)	0.0025 (6)	0.0030 (6)	0.0009 (5)
C6	0.0187 (7)	0.0182 (6)	0.0187 (7)	0.0015 (6)	0.0034 (6)	−0.0031 (6)
C7	0.0140 (7)	0.0158 (6)	0.0220 (7)	0.0021 (6)	0.0038 (6)	0.0026 (5)
C31	0.0127 (7)	0.0167 (6)	0.0193 (7)	−0.0005 (5)	0.0008 (5)	0.0002 (5)
C71	0.0172 (7)	0.0168 (6)	0.0294 (8)	−0.0010 (6)	0.0047 (6)	0.0055 (6)
O1W	0.0263 (7)	0.0294 (6)	0.0394 (7)	−0.0032 (5)	0.0118 (5)	0.0012 (5)

### Geometric parameters (Å, º)

**Table:**

O31—C31	1.2580 (17)	C2—C6	1.511 (2)
O32—C31	1.2341 (16)	C3—C4	1.5556 (19)
O71—C71	1.2174 (19)	C3—C31	1.5498 (19)
O72—C71	1.3180 (18)	C4—C7	1.5615 (19)
O72—H72	0.94 (3)	C4—C5	1.5407 (18)
O1W—H11W	0.90 (2)	C5—C6	1.520 (2)
O1W—H12W	0.93 (2)	C7—C71	1.509 (2)
N31—C3	1.5026 (17)	C1—H1	0.9800
N31—H31A	0.94 (2)	C2—H2	0.9800
N31—H31B	0.91 (2)	C4—H4	0.9800
N31—H31C	0.874 (18)	C5—H5A	0.9700
C1—C7	1.510 (2)	C5—H5B	0.9700
C1—C6	1.5095 (19)	C6—H6	0.9800
C1—C2	1.525 (2)	C7—H7	0.9800
C2—C3	1.5125 (19)		
			
C71—O72—H72	116.7 (16)	C1—C7—C4	97.18 (11)
H11W—O1W—H12W	109 (2)	C4—C7—C71	110.69 (11)
H31A—N31—H31B	110.8 (18)	O31—C31—O32	125.46 (13)
C3—N31—H31C	114.7 (11)	O31—C31—C3	114.95 (11)
C3—N31—H31A	109.1 (12)	O32—C31—C3	119.58 (12)
C3—N31—H31B	110.1 (10)	O71—C71—C7	125.36 (13)
H31B—N31—H31C	103.1 (15)	O72—C71—C7	115.84 (12)
H31A—N31—H31C	108.9 (16)	O71—C71—O72	118.79 (14)
C6—C1—C7	106.96 (12)	C2—C1—H1	122.00
C2—C1—C7	106.99 (11)	C6—C1—H1	122.00
C2—C1—C6	59.72 (9)	C7—C1—H1	122.00
C3—C2—C6	106.15 (11)	C1—C2—H2	122.00
C1—C2—C6	59.64 (9)	C3—C2—H2	122.00
C1—C2—C3	107.21 (11)	C6—C2—H2	122.00
N31—C3—C4	111.41 (10)	C5—C4—H4	117.00
N31—C3—C31	106.12 (11)	C7—C4—H4	117.00
N31—C3—C2	112.81 (11)	C3—C4—H4	117.00
C2—C3—C31	116.96 (11)	C4—C5—H5A	112.00
C4—C3—C31	112.14 (11)	C6—C5—H5A	112.00
C2—C3—C4	97.41 (10)	C6—C5—H5B	112.00
C3—C4—C5	100.95 (10)	C4—C5—H5B	112.00
C5—C4—C7	101.06 (11)	H5A—C5—H5B	110.00
C3—C4—C7	101.48 (10)	C2—C6—H6	122.00
C4—C5—C6	96.91 (10)	C5—C6—H6	122.00
C2—C6—C5	107.50 (11)	C1—C6—H6	122.00
C1—C6—C5	107.01 (11)	C4—C7—H7	111.00
C1—C6—C2	60.64 (9)	C71—C7—H7	111.00
C1—C7—C71	116.11 (12)	C1—C7—H7	111.00
			
C6—C1—C2—C3	−98.97 (12)	C2—C3—C4—C7	−50.96 (11)
C7—C1—C2—C3	1.10 (14)	C31—C3—C4—C5	−70.29 (13)
C7—C1—C2—C6	100.07 (12)	C31—C3—C4—C7	−174.09 (10)
C2—C1—C6—C5	100.87 (12)	N31—C3—C31—O31	34.29 (16)
C7—C1—C6—C2	−100.12 (12)	N31—C3—C31—O32	−146.79 (13)
C7—C1—C6—C5	0.76 (15)	C2—C3—C31—O31	161.14 (12)
C2—C1—C7—C4	−31.88 (13)	C2—C3—C31—O32	−19.94 (19)
C2—C1—C7—C71	−149.16 (12)	C4—C3—C31—O31	−87.56 (14)
C6—C1—C7—C4	30.86 (13)	C4—C3—C31—O32	91.36 (15)
C6—C1—C7—C71	−86.42 (14)	C3—C4—C5—C6	−51.88 (12)
C1—C2—C3—N31	−86.65 (13)	C7—C4—C5—C6	52.27 (12)
C1—C2—C3—C4	30.35 (12)	C3—C4—C7—C1	51.65 (12)
C1—C2—C3—C31	149.86 (11)	C3—C4—C7—C71	173.10 (11)
C6—C2—C3—N31	−149.18 (11)	C5—C4—C7—C1	−52.08 (12)
C6—C2—C3—C4	−32.19 (12)	C5—C4—C7—C71	69.37 (13)
C6—C2—C3—C31	87.33 (14)	C4—C5—C6—C1	−32.48 (14)
C1—C2—C6—C5	−100.06 (12)	C4—C5—C6—C2	31.34 (13)
C3—C2—C6—C1	100.80 (12)	C1—C7—C71—O71	6.7 (2)
C3—C2—C6—C5	0.74 (14)	C1—C7—C71—O72	−174.76 (12)
N31—C3—C4—C5	170.94 (11)	C4—C7—C71—O71	−102.86 (16)
N31—C3—C4—C7	67.13 (13)	C4—C7—C71—O72	75.74 (15)
C2—C3—C4—C5	52.85 (11)		

### Hydrogen-bond geometry (Å, º)

**Table:**

*D*—H···*A*	*D*—H	H···*A*	*D*···*A*	*D*—H···*A*
O1*W*—H11*W*···O71^i^	0.90 (2)	2.02 (2)	2.9161 (16)	176 (2)
O1*W*—H12*W*···O31^ii^	0.93 (2)	1.77 (2)	2.6792 (16)	168 (2)
N31—H31*A*···O31^ii^	0.94 (2)	1.87 (2)	2.7712 (17)	161 (2)
N31—H31*B*···O71^iii^	0.91 (2)	2.29 (2)	3.1261 (17)	153.0 (15)
N31—H31*B*···O72^iii^	0.91 (2)	2.27 (2)	3.0720 (17)	147.6 (15)
N31—H31*C*···O32^iv^	0.874 (18)	1.925 (17)	2.7769 (16)	164.4 (17)
O72—H72···O1*W*	0.94 (3)	1.60 (3)	2.5282 (16)	174 (3)
C5—H5*A*···O32	0.97	2.51	3.0865 (19)	118
C6—H6···O72^v^	0.98	2.53	3.1105 (17)	118
C7—H7···N31	0.98	2.56	2.9097 (19)	101
C7—H7···O32^iv^	0.98	2.35	3.2678 (17)	155

Symmetry codes: (i) −*x*, *y*−1/2, −*z*+1/2; (ii) −*x*+1, −*y*, −*z*; (iii) *x*+1, *y*, *z*; (iv) −*x*+1, *y*−1/2, −*z*+1/2; (v) −*x*, *y*+1/2, −*z*+1/2.
